# Exploring the Regulatory Mechanism of Modified Huanglian Maidong Decoction on Type 2 Diabetes Mellitus Biological Network Based on Systematic Pharmacology

**DOI:** 10.1155/2021/1768720

**Published:** 2021-07-12

**Authors:** Qi He, Tianqing Zhang, Bing Jin, Yonghe Wu, Jiamin Wu, Pu Gao, Shiwei Wu

**Affiliations:** ^1^People's Hospital of Ningxiang City, Ningxiang City, Hunan Province, China; ^2^Graduate College, University of South China, Hengyang, Hunan Province, China; ^3^Xiyuan Hospital, China Academy of Chinese Medical Sciences, Beijing, China; ^4^Department of Traditional Chinese Medicine, The Eighth Affiliated Hospital, Sun Yat-sen University, Shenzhen, Guangdong Province, China

## Abstract

**Objective:**

To explore the mechanism of modified Huanglian Maidong decoction (Maidong-Sanqi-Huanglian Compounds, MSHCs) intervention in type 2 diabetes mellitus (T2DM).

**Method:**

This study used PubChem and SciFinder to collect the molecular structure of MSHCs, used PharmMapper to predict the potential targets of MSHC, and combined them with the T2DM gene to construct MSHC-T2DM protein-protein interaction (PPI) network. The plugin MCODE in Cytoscape 3.7.1 was then used to perform cluster analysis on the MSHC-T2DM PPI network. The genes and targets were input into DAVID for Gene Ontology (GO) and pathway enrichment analysis. Finally, animal experiments were performed to verify the therapeutic effect of MSHC on T2DM.

**Results:**

Several T2DM-related targets, clusters, signaling pathways, and biological processes are found. The experimental results showed that compared with the blank group, the content of fasting blood glucose (FBG) in the model group was higher (*P* < 0.01). Compared with the model group, the content of FBG decreased and the insulin level increased in the MSHC medium-dose (0.15 g/kg) and high-dose (0.45 g/kg) groups and metformin group after 4 weeks of drug administration (*P* < 0.05). MSHC can also improve blood liquid levels and inflammatory factor levels (*P* < 0.05).

**Conclusion:**

MSHC may achieve therapeutic effects through regulating the T2DM-related targets, biological processes, and pathways, such as insulin resistance, energy metabolism, oxidative stress, and inflammation, found in this research.

## 1. Introduction

Diabetes is a series of metabolic disorder syndrome caused by islet dysfunction, insulin resistance, etc., and often accompanied by a variety of complications [1, 2]. At present, the global adult diabetic patients (aged 20–79) have increased from 151 million in 2000 to 425 million in 2017, a nearly double increase [3]. Due to urbanization, population aging, obesity, and other reasons, the number of patients with diabetes continues to grow rapidly. It is estimated that by 2045, diabetes patients may reach 629 million [4]. So far, the allopathic medicine was usually based on injection of insulin or oral glucose-lowering drugs such as metformin and acarbose. However, due to the expensive prices and side effects of these drugs, patient compliance has gradually decreased [5, 6]. Traditional Chinese medicine (TCM) has a unique understanding of diabetes as a systemic endocrine disease. Meanwhile, TCM can well intervene and regulate diabetes from the perspective of the overall system [7, 8]. Herbal medicine has gradually attracted attention due to its mildness, multiple targets, and fewer adverse reactions [9, 10].

Huanglian Maidong decoction comes from *Dou Ke Lei Bian* Volume III (Written by Zhai Liang in the Ming Dynasty), which is composed of *Coptis chinensis* Franch. (Copidis*Rhizoma*) and *Ophiopogon japonicus* (L. f.) Ker GawL (*Ophiopogon japonicus*). The herbal formula has been used in ancient times to treat Xiaoke Bing, namely, diabetes, and its effect is exact. Our previous research showed that modified Huanglian Maidong decoction (also called *Ophiopogon japonicus*-*Panax notoginseng* (Burk.) F. H. Chen ex C. Chow-*Copidis Rhizoma* Compounds (Maidong-Sanqi-Huanglian Compounds, MSHCs)) can significantly improve fasting blood glucose and glycated hemoglobin levels in patients with type 2 diabetes mellitus (T2DM) [11]. We hope to clarify its mechanism for treating T2DM through new strategies.

Due to the complex compounds and targets of TCM, the classical pharmacology presents difficulties analyzing its complex intervention mechanisms [12]. Hopkins proposed the concept of “network pharmacology” in 2007 and gave new ideas for TCM research, especially in combination with multidirectional pharmacology [13–15]. Network pharmacology can study the therapeutic mechanism of drugs from the holistic and systemic aspects of the interaction between Chinese medicine compounds, targets, and diseases. This is particularly suitable for studying the role of TCM multi-components-multi-targets and is conducive to revealing its complex mechanism [16–19]. Therefore, this study will combine the methods of experimental pharmacology and network pharmacology to explore the therapeutic mechanism of MSHC on T2DM.

## 2. Materials and Methods

### 2.1. MSHC Acquisition

Previous studies have shown that the main components of MSHC are *Panax notoginseng* saponins (PNS), *Ophiopogon* polysaccharide, and *Coptis* alkaloids. [20]. The main monomer component in *Ophiopogon* polysaccharide is Polysaccharide MGD-1 [21]. The main monomer components in *Coptis* alkaloids are Palmatine, Jatrorrhizine, Epiberberine, Coptisine, Columbamine, and Berberine [22]. The main monomer components in PNS are Ginsenoside Rb1, Ginsenoside Ro, Ginsenoside Rg1, Ginsenoside Rh2, Notoginsenoside R1, Ginsenoside Rh1, Pseudoginsenoside F11, Ginsenoside Re, Ginsenoside K, Ginsenoside Rb3, Ginsenoside Rg3, and Ginsenoside Rg5 [23]. The molecular structure of these components was searched in PubChem (https://pubchem.ncbi.nlm.nih.gov/) and SciFinder (http://scifinder.cas.org). Then, the structures were drawn by ChemBioDraw 14, a chemical drawing software application that provides scientists with a complete and easy-to-use drawing solution, and saved in mol2 format.

### 2.2. MSHC's Potential Target Prediction and T2DM Gene Collection

The mol2 files of all compounds were input into PharmMapper (http://lilab-ecust.cn/pharmmapper/) to predict the potential targets of MSHC [24]. The reverse docking prediction results for each compound were downloaded and arranged in descending order according to the docking score (*Z*) values. The first 300 targets of each compound were collected for subsequent research. The GeneCards database (http://www.genecards.org/) and the OMIM database (http://www.ncbi.nlm.nih.gov/omim) were used to collect T2DM-related genes [25, 26].

The search function of UniProt KB (https://www.uniprot.org/uniprot/) was utilized to import the PDB ID number of the MSHC target, with the species restricted to human. After the above operation, the “official symbol” of the MSHC target is obtained. The MSHC targets are shown in Table S1 and the T2DM genes are shown in Table S2 (see supplementary materials).

### 2.3. Network Construction and Analysis

The data of protein-protein interaction (PPI) were obtained from String 11.0 (http://string-db.org/) [27] and InAct (http://www.ebi.ac.uk/intact/) [28]. The T2DM gene, MSHC and potential targets, and PPI data were imported into Cytoscape 3.7.1 software (https://cytoscape.org/) for network construction [29].

The networks were analyzed by the plugin molecular complex detection (MCODE) to obtain cluster [14, 15, 17–19]. MCODE can be used to discover closely related regions in the PPI network, and these regions may represent molecular complexes. According to the given parameters, dense regions are separated, which has advantages over other cluster methods because other methods rarely consider the rest of the network.

### 2.4. Gene Ontology (GO) and Pathway Enrichment Analysis

DAVID (https://david.ncifcrf.gov/summary.jsp, Version 6.8) provides systematic comprehensive biological function annotation information for large-scale genes or proteins, which can find the most significantly enriched biological annotations [30]. The targets and genes were imported into the DAVID database, the Identifier was set to official gene symbol, the List Type was set to GeneList, and the species was restricted to “*Homo sapiens*.” Then, the Gene Ontology (GO) analysis and KEGG pathway analysis of MSHC targets and T2DM genes can be performed.

### 2.5. Experimental Materials

#### 2.5.1. Animals

Seventy-six (76) Specific Pathogen Free (SPF) grade Sprague-Dawley (SD) rats (half males and half females) (9 weeks old, body weight 190–210 g) were provided by Hunan Slack Jingda Experimental Animal Co., Ltd. The animal license number is SCXK (Xiang) 2016-0017. All rats were given conventional animal feed and water, and the feed was full-scale solid pellet feed for breeding rats (contains crude protein 18%, crude fat 5%, crude fiber 5%, crude ash 8%, amino acid 9.23%, calcium 1.00%, phosphorus 0.85%, potassium 0.55%, sodium 0.25%, vitamin A 17 IU/g, vitamin *D* 4 U/g, vitamin *E* 45 ppm, vitamin K 2.0 ppm, vitamin B12 0.08 ppm, etc.) (Liaoning Changsheng Biotechnology Co., Ltd.). All animals' care and experimental procedures were approved by the Ethical Committee on Animal Research at Liaoning Basic Medical Research Institute and were in accordance with the National Institute of Health's Guide for the Care and Use of Laboratory Animals (Approval No. LUTCM2016020023).

#### 2.5.2. Intervention Drugs

PNS was provided by Hunan Warner Pharmaceutical Co., Ltd. (DT-2311). Ophiopogon polysaccharide and *Coptis* alkaloids were provided by Shanghai Yiben Biomedical Technology Co., Ltd. (MD00065). The proportion of MSHC is 40% *Ophiopogon* polysaccharide, 30% *Coptis* alkaloids, and 30% *Panax notoginseng* saponins). Metformin tablets (Shandong Boshan Pharmaceutical Co., Ltd., batch number 20130812) were formulated with distilled water to prepare a solution with the required concentration.

#### 2.5.3. Main Reagents and Instruments

Streptozotocin (STZ) (Sigma, lot number: S0213); 125I-insulin radioimmunoassay kit (Beijing Atomic High-Tech Nuclear Technology Application Co., Ltd.); Sannuo SXT-1 fast blood glucose tester (Changsha Sannuo Biosensor Technology Co., Ltd.). Insulin, total cholesterol (TC), triglycerides (TG), high density lipoprotein cholesterol (HDL-C), and low density lipoprotein cholesterol (LDL-C) detection kits (Nanjing Jiancheng Institute of Bioengineering, batch numbers: 20160119, 201701121, 201700124, 20170811, and 20170223). Rabbit anti-mouse antibodies TNF-*α*, IL-6, and MCP-1 ELISA kit (BPB Biomedical, batch numbers: BA001023, BA000101, and BA00017). Blood Glucose Test Strips (Cat. No. AB-103; Wuding Biotechnology Co., Ltd.). Fully automatic biochemical analyzer (Hitachi 7180 type, Hitachi Inc.); Labsystems Multiskan MS 1353 microplate reader and horizontal electrophoresis tank (Shanghai Experimental Instrument Factory Co., Ltd.).

### 2.6. Experimental Methods

#### 2.6.1. Animal Modeling, Grouping, and Intervention

According to the method of reference [31], the diabetic rat model was made by intraperitoneal injection of STZ. One week after the adaptive feeding of rats, 10 rats were randomly selected as the blank group according to the random number table method, and the rest were intraperitoneally injected with STZ 0.055 mg/gbody weight. After 1 week, the fasting blood glucose (FBG) of the rats was examined. Rats with FBG ≥11.1 mmol/L were considered as a successful model. Rats with successful modeling were randomly assigned to 5 groups according to the random number table method: (1) model group (10 rats); (2) metformin group (10 rats); (3) MSHC high-dose group (10 rats); (4) MSHC medium-dose group (10 rats); (5) MSHC low-dose group (10 rats). The male to female ratio was 1 : 1 in all 6 groups.

MSHC high-, medium-, and low-dose groups were administered intragastrically with MSHC at 0.45, 0.15, and 0.05 g/kg, respectively. The metformin group was given oral metformin 0.05 mg/kg intragastrically. The model group and the blank group were intragastrically administered with an equal volume of saline solution (0.9%). The intragastric administration volume in each group was 10 mL/kg.

#### 2.6.2. Detection of FBG, Insulin, and Blood Liquid Levels

Tail vein blood was taken 1 d before and 4 weeks after the administration, and FBG was detected by Blood Glucose Test Strips (glucose oxidase method). The FBG was detected strictly according to the kit instructions. After 4 weeks of drug administration, the rats were euthanized and blood were collected under 2% sodium pentobarbital anesthesia. The blood was allowed to stand for 60 minutes and centrifuged at 1000 g/min for 15 minutes to separate the serum. The TC, TG, HDL-C, and LDL-C levels were detected by 7180 automatic biochemical analyzer. The insulin level was detected by radioimmunoassay. The operation was strictly performed according to the kit instructions. Insulin and resistance index are calculated based on fasting blood glucose and fasting insulin, and the calculation formula is insulin resistance index (IRI) = (fasting blood glucose × fasting insulin)/22.5.

#### 2.6.3. Determination of Inflammatory Factor Levels in Serum

Serum TNF-*α*, IL-6, and MCP-1 levels were detected strictly in accordance with the ELISA kit instructions.

#### 2.6.4. Pathological Observation

After blood collection, the pancreas was harvested, fixed in 4% paraformaldehyde solution for 12 h, embedded in conventional paraffin, sectioned, and stained with hematoxylin and eosin (H&E). The pathological changes of the islet tissue were observed under an optical microscope.

### 2.7. Statistical Analysis

The SPSS22.0 statistical software was used for analysis. The experimental data are expressed as mean ± SD. The data consistent with normality and homogeneity of variance were analyzed by analysis of variance (ANOVA). For multiple comparisons, the comparison between the two groups was performed using the LSD method. Non-parametric analysis was used for non-conformances. *P* < 0.05 indicates that the difference is statistically significant.

## 3. Results

### 3.1. T2DM Genes and MSHC Potential Targets

**Figure 1:**
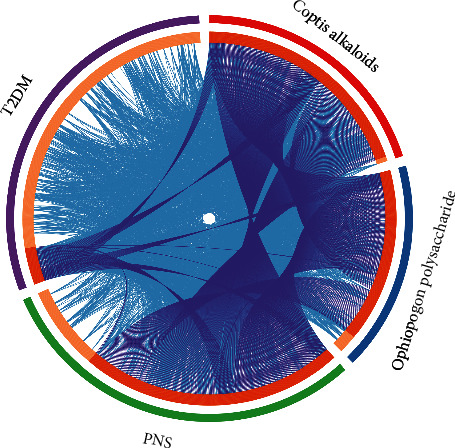
The circle plot of potential targets and T2DM gene distribution.

A total of 5719 T2DM genes were obtained. The genes with relevance score ≥9 were selected to construct the MSHC-T2DM PPI network. After the potential target protection, totally 420 human potential targets are collected (Figure 1). In the outer circle, purple, red, blue, and green represent T2DM genes, *Ophiopogon* polysaccharide targets, *Coptis* alkaloids targets, and PNS targets, respectively. In the inner circle, the greater the number of purple links and the longer the dark orange arc, the greater the number of overlapping genes. The blue link indicates the amount of functional overlap between the input target lists.

### 3.2. MSHC-T2DM PPI Network Analysis

#### 3.2.1. MSHC-T2DM PPI Network

The MSHC targets and T2DM genes were input into String to collect their PPI data. Then, the MSHC targets, T2DM genes, and their PPI data were utilized to build the MSHC-T2DM PPI network by Cytoscape 3.7.1.

**Figure 2:**
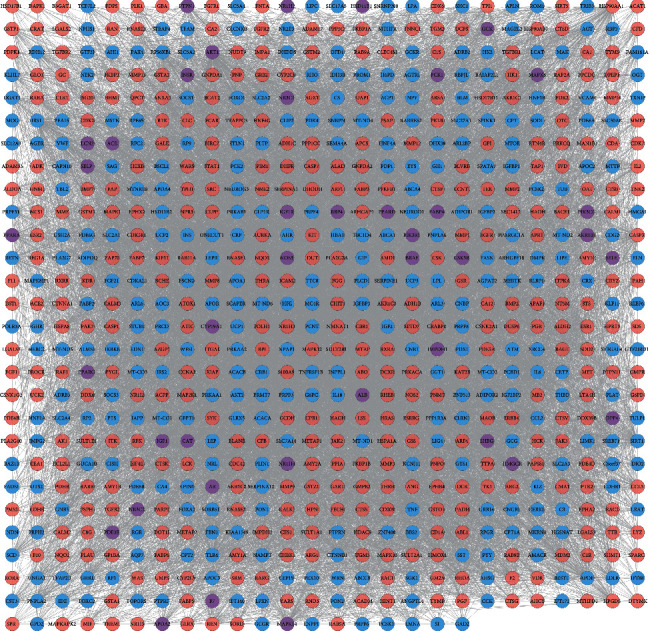
MSHC-T2DM PPI network (blue circle stands for T2DM gene; pink circle stands for MSHC target; purple circle stands for MSHC-T2DM target).

This network is composed of 721 nodes (368 MSHC target nodes, 313 T2DM gene nodes, and 40 MSHC-T2DM target nodes) and 13908 edges. The targets in the network are arranged in descending order according to degree. The top 20 are (1) MSHC targets: EGFR (180 edges), MAPK1 (179 edges), SRC (173 edges), CASP3 (155 edges), MMP9 (144 edges), and HSP90AA1 (139 edges); (2) DCM genes: INS (354 edges), IL6 (241 edges), TNF (211 edges), LEP (170 edges), SIRT1 (145 edges), ADIPOQ (145 edges), MTOR (140 edges), and IRS1 (138 edges); (3) MSHC-DCM targets: ALB (279 edges), AKT1 (271 edges), IGF1 (174 edges), PPARG (156 edges), CAT (155 edges), and MAPK8 (155 edges) (Figure 2).

#### 3.2.2. Clusters of MSHC-T2DM PPI Network

**Figure 3:**
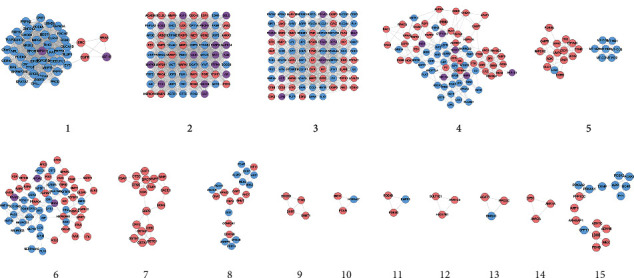
Clusters of MSHC-T2DM PPI network (blue circle stands for T2DM gene; pink circle stands for MSHC target; purple circle stands for MSHC-T2DM target).

The MSHC-T2DM PPI network was analyzed by MCODE and returns 15 clusters (Figure 3). Then, the genes and targets were input into DAVID for GO enrichment analysis.

Cluster 1 is associated with calcium metabolism, ERBB2, and Wnt signaling. Cluster 2 is related to glucose metabolism, insulin and its related signaling pathways, vascular smooth muscle proliferation, and lipid metabolism. Cluster 3 is involved in hypoxia, glucose homeostasis, insulin resistance, adiponectin-related pathways, inflammation, and energy metabolism. Cluster 4 is related to cholesterol metabolism, glucose metabolism, lipid metabolism, and MAPK cascade. Cluster 6 is involved in glucose homeostasis and insulin secretion. Cluster 7 is related to redox. Cluster 15 is associated with lipid metabolism, glucose metabolism, and energy metabolism. Cluster 10 failed to return any human biological processes. Clusters 5, 8, 9, 11, 12, 13, and 14 did not return any T2DM-related biological processes. The details are shown in Table S3.

**Figure 4:**
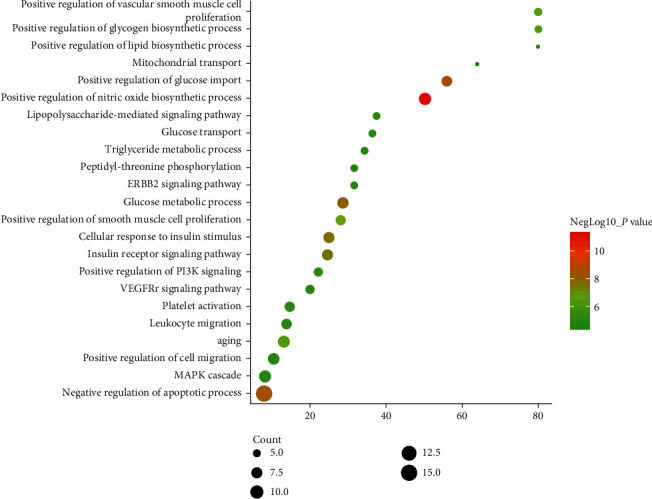
Bubble chart of biological processes of cluster 2.

Since the biological process of cluster 2 is representative, the main biological process in cluster 2 is selected as an example for demonstration (Figure 4).

#### 3.2.3. Signaling Pathways of MSHC-T2DM PPI Network

**Figure 5:**
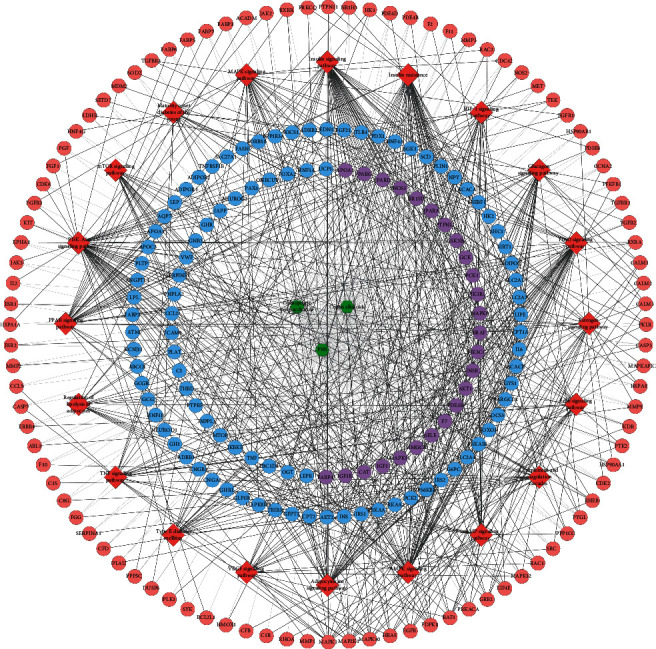
Signaling pathways of MSHC-T2DM PPI network (red diamond stands for signaling pathways; green hexagon stands for herb; blue circle stands for T2DM gene; pink circle stands for MSHC target; purple circle stands for MSHC-T2DM target; light lines stand for relationships among herbs and targets; dark lines stand for relationships among signaling pathways and targets).

**Figure 6:**
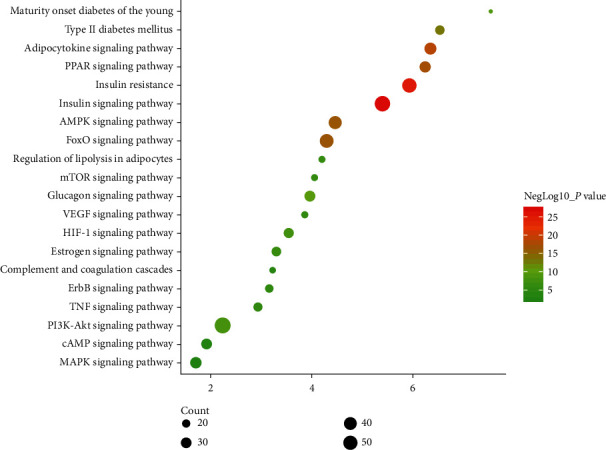
Bubble chart of signaling pathways.

All the interacting targets in the MSHC-T2DM PPI network were introduced into DAVID for pathway enrichment analysis, and totally 20 signaling pathways were obtained (Figure 5). These signaling pathways are arranged according to the degree of enrichment (negative correlation with *P* value) and count from large to small. The top 10 are insulin signaling pathway (*P* = 9.18^*∗*^ 10^−28, count = 57), insulin resistance (*P* = 4.88^*∗*^ 10^−26, count = 49), adipocytokine signaling pathway (*P* = 3.26^*∗*^ 10^−19, count = 34), PPAR signaling pathway (*P* = 7.97^*∗*^ 10^−18, count = 32), FoxO signaling pathway (*P* = 5.25^*∗*^ 10^−17, count = 44), AMPK signaling pathway (*P* = 6.25^*∗*^ 10^−17, count = 42), type II diabetes mellitus (*P* = 6.38^*∗*^ 10^−14, count = 24), glucagon signaling pathway (*P* = 1.04^*∗*^ 10^−10, count = 30), maturity onset diabetes of the young (*P* = 7.88^*∗*^ 10^−10, count = 15), and PI3K-Akt signaling pathway (*P* = 4.51^*∗*^ 10^−09, count = 59) (Figure 6). The details are shown in Table S4.

### 3.3. Effect of MSHC on Body Weight in Rats

**Figure 7:**
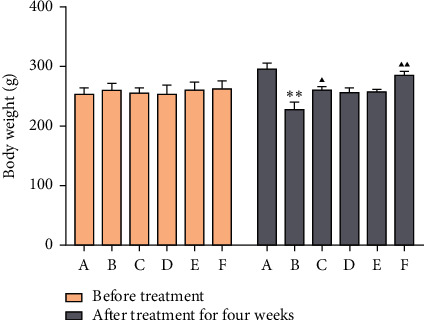
Effect of MSHC on body weight in rats (A: blank control group; B: model group; C: MSHC high-dose group; D: MSHC medium-dose group; E: MSHC low-dose group; F: metformin group; compared with the blank group, ^*∗∗*^*P* < 0.01; compared with the model group, ▲*P* < 0.05, ▲▲*P* < 0.05).

The weight difference of rats in each group before intervention was not significant (*P* > 0.05). After 4 weeks of intervention, compared with the model group and each treatment group, the differences were significant (*P* < 0.01). It shows that MSHC and metformin can effectively inhibit the weight loss trend of type 2 diabetic rats (Figure 7).

### 3.4. Effect of MSHC on FBG in Rats

**Figure 8:**
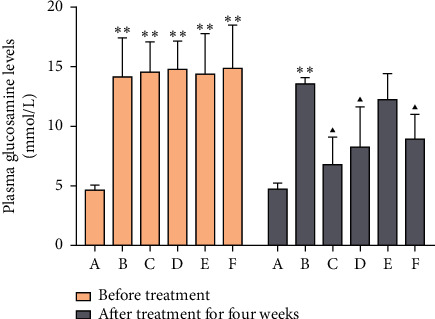
Comparison of FBG content before and after administration (A: blank control group; B: model group; C: MSHC high-dose group; D: MSHC medium-dose group; E: MSHC low-dose group; F: metformin group; compared with the blank group, ^*∗∗*^*P* < 0.01; compared with the model group, ▲*P* < 0.05).

Compared with the blank group, the FBG in the model group was significantly increased before and after 4 weeks (*P* < 0.01). Compared with the model group, FBG was significantly reduced after 4 weeks of administration in the MSHC medium- and high-dose groups and metformin group (*P* < 0.05) (Figure 8).

### 3.5. Effects of MSHC on Serum Insulin Level and IRI in Rats

**Figure 9:**
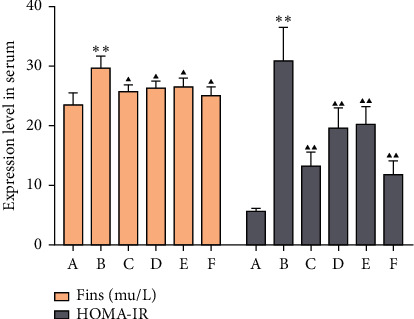
Comparison of serum insulin level and IRI in rats (FINS: fasting insulin; HOMA-IR: homeostasis model assessment-insulin resistance; A: blank control group; B: model group; C: MSHC high-dose group; D: MSHC medium-dose group; E: MSHC low-dose group; F: metformin group; compared with the blank group, ^*∗∗*^*P* < 0.01; compared with the model group, ▲*P* < 0.05, ▲▲*P* < 0.01).

Compared with the blank group, the insulin level of the model group was significantly reduced, and the IRI was significantly increased (*P* < 0.01). Compared with the model group, the insulin level in the MSHC medium- and high-dose groups and metformin group was significantly increased, and the IRI was significantly decreased (*P* < 0.01) (Figure 9).

### 3.6. Effects of MSHC on Serum TC, TG, HDL-C, and LDL-C in Rats

**Figure 10:**
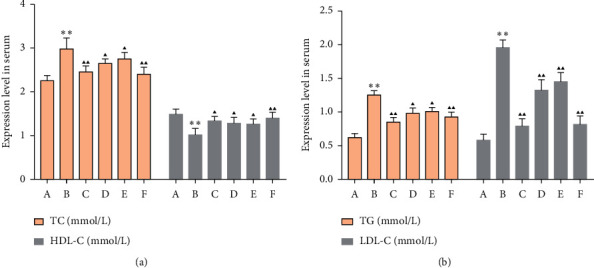
Effects of MSHC on serum TC, TG, HDL-C, and LDL-C in rats: (a) TC and HDL-C levels; (b) TG and LDL-C levels (A: blank control group; B: model group; C: MSHC high-dose group; D: MSHC medium-dose group; E: MSHC low-dose group; F: metformin group; compared with the blank group, ^*∗∗*^*P* < 0.01; compared with the model group, ▲*P* < 0.05, ▲▲*P* < 0.01).

Compared with the blank group, the TC, TG, and LDL-C levels of the model group increased, while HDL-C level decreased (*P* < 0.05). Compared with the model group, the TC, TG, and LDL-C levels in the MSHC medium- and high-dose groups and metformin group were significantly decreased, while HDL-C increased (*P* < 0.01) (Figure 10). It shows that MCSH can reduce serum TC, TG, and LDL-C, increase HDL-C, and improve lipid metabolism in T2DM rats.

### 3.7. Effects of MSHC on Serum Inflammatory Factor in Rats

**Figure 11:**
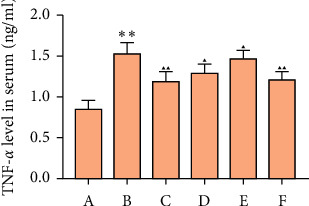
Effects of MSHC on serum TNF-*α* in rats (A: blank control group; B: model group; C: MSHC high-dose group; D: MSHC medium-dose group; E: MSHC low-dose group; F: metformin group; compared with the blank group, ^*∗∗*^*P* < 0.01; compared with the model group, ▲*P* < 0.05, ▲▲*P* < 0.01).

**Figure 12:**
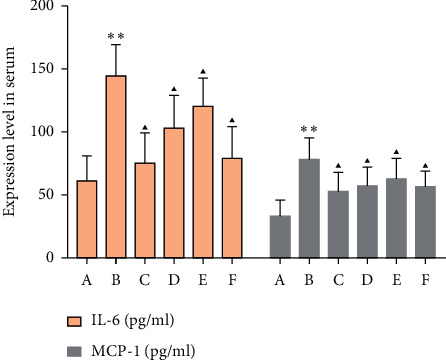
Effects of MSHC on serum IL-6 and MCP-1 in rats (A: blank control group; B: model group; C: MSHC high-dose group; D: MSHC medium-dose group; E: MSHC low-dose group; F: metformin group; compared with the blank group, ^*∗∗*^*P* < 0.01; compared with the model group, ▲*P* < 0.05).

Compared with the blank group, the TNF-*α*, IL-6, and MCP-1 of the model group increased (*P* < 0.05). Compared with the model group, the TNF-*α*, IL-6, and MCP-1 in the MSHC medium- and high-dose groups and metformin group were significantly decreased (*P* < 0.05 or 0.01) (Figures 11 and 12).

### 3.8. Pathological Changes

**Figure 13:**
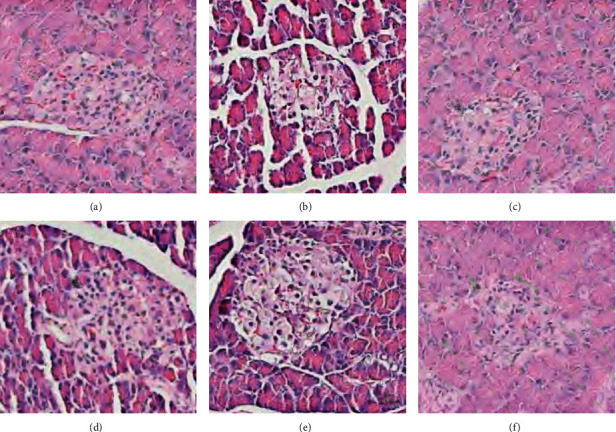
Pathological changes (HE staining, 400×): (a) blank control group; (b) model group; (c) MSHC high-dose group; (d) MSHC medium-dose group; (e) MSHC low-dose group; (f) metformin group.

The islet *β* cells of the blank control group are tightly arranged, the size is the same, the nuclear chromatin is clear, and the cytoplasmic boundary is clear; the islet *β* cells of the model group are loosely arranged, the cell sizes are different, and the cytoplasmic vacuolation is serious. Compared with the model group, the pancreatic islet *β* cells in the MSHC medium- and low-dose groups are less loosely arranged and cytoplasmic vacuolation is less. The morphological structure of pancreatic islet *β* cells in MSHC high-dose group and metformin group showed that the cell size was more uniform and the arrangement and the cytoplasm was more uniform. It shows that MSHC has a protective effect on the islet *β* cells of T2DM rats (Figure 13).

## 4. Discussion

Insulin resistance is the main pathological basis of T2DM, which refers to the decrease of insulin utilization rate and decrease of the body's sensitivity to insulin [2, 32]. Current research suggests that the main cause of insulin resistance is an increase in inflammatory cytokines, which interferes with the normal phosphorylation of IRS in insulin signaling pathway and blocks a series of cascade amplification reactions activated by downstream signals, thereby affecting physiological functions such as insulin production and transport [4, 33, 34].

Inflammatory cytokines and other factors can interfere with the normal insulin signaling pathway and activate the insulin resistance signaling pathway, leading to insulin resistance and T2DM [35, 36]. Currently, the insulin-related signaling pathways those have been deeply studied are PI3K signaling pathway and Ras-RafMAPK signaling pathway [37, 38]. Meanwhile,insulin resistance-related signaling pathways include c-Jun amino terminal kinase (JNK) signaling pathway, NF-*κ*B signaling pathway, SOCS-3 signaling pathway, INOS-NO signaling pathway, PKC signaling pathway, and other pathways related to PTP-1 B and glycogen synthase 3 [39–43]. The essence of the insulin signaling pathway and insulin resistance signaling pathway is the result of joint effects of inflammatory cytokines, transcription factors, phosphorylation of various protein kinases, and protein-protein interactions [42, 43]. In this study, through the T2DM gene collection, MSHC target prediction, and the construction and analysis of networks such as the MSHC-T2DM PPI network, the potential regulatory mechanism of MSHC on T2DM disease networks was discovered. For example, we found that MSHC can regulate various functional modules (namely, cluster) in the T2DM's complex bionetwork. Each module contains many biological processes, suggesting that MSHC may regulate the entire module by regulating these biological processes such as GO:0045429, GO:0046326, GO:0043066, GO:0006006, GO:0032869, GO:0008286, GO:0048661, GO:1904707, GO:0045725, GO:0007568, GO:0014068, GO:0030168, GO:0031663, GO:0015758, GO:0030335, GO:0050900, GO:0048010, GO:0000165, GO:0006641, GO:0046889, GO:0018107, GO:0038128, and GO:0006839. More interestingly, this study revealed the regulation effect of MSHC on each module in the entire complex network of T2DM at the system level, which provided a new research direction for future research on MSHC treatment of T2DM.

Through the enrichment of targets and genes in the MSHC-T2DM PPI network, it was found that the potential mechanisms of MSHC treatment for T2DM focus on insulin resistance, energy metabolism, oxidative stress, and inflammation. The insulin-related signaling pathways are insulin resistance and insulin signaling pathways. The energy metabolism-related signaling pathways are the adipocytokine signaling pathway and the PPAR signaling pathway. The oxidative stress-related signaling pathways include FoxO signal pathway, AMPK signal pathway, and so on. The inflammatory-related signaling pathways include PI3K-Akt, Wnt, TGF-*β*, TNF signaling pathways, and so on. These signaling pathways mediate all aspects of the development of T2DM. They are both independent and connected to each other and form a close regulatory network through extensive interaction to jointly promote the development of T2DM.

Current research also shows that *Coptis* alkaloids can improve insulin resistance, promote insulin secretion, inhibit gluconeogenesis in the liver, stimulate glycolysis of surrounding tissue cells, reduce intestinal absorption of glucose, and regulate lipid metabolism by inhibiting oxidative stress and inflammation in a variety of tissues including the liver, adipose tissue, kidney, and pancreas [44–48]. Its specific mechanism mainly involves a variety of cell kinases and signaling pathways, such as the AMPK, MAPK, Nrf2, and NF-*κ*B signaling. *Ophiopogon* polysaccharide can reduce pancreatic islet damage in diabetic mice induced by alloxan [49]. *Ophiopogon* polysaccharide can also reduce FBG levels and increase oral glucose tolerance in diabetic mice [50]. It can also reduce insulin resistance by regulating the InsR/IRS-1/PI3K/Akt/GSK-3/Glut-4 signaling pathways [51, 52]. The results of these studies also illustrate the accuracy of target prediction based on the pharmacophore-based response molecule docking technology.

## 5. Conclusion

In summary, this study used TCMSP, GeneCards, Cytoscape, and other databases/tools to study the mechanism of MSHC for T2DM from multiple aspects (biological processes, pathways, and so on). The results of animal pharmacological experiments show that MSHC can reduce FBG and increase basal insulin levels in diabetic rats; MSHC can also improve blood liquid levels and inflammatory factor levels.

## Data Availability

The data used to support the findings of this study are included within the article and the supplementary information files.

## Supplementary Materials

Supplementary Materials Table S1: potential targets for each compound. Table S2: type 2 diabetes mellitus genes. Table S3: enrichment analysis of clusters based on Gene Ontology (GO) annotation. Table S4: pathway enrichment analysis.
